# Firearm Storage Practices Among Dementia Caregivers: Findings From the BRFSS

**DOI:** 10.1093/geroni/igaf122.1085

**Published:** 2025-12-31

**Authors:** Jiaming Liang, Alexander Testa

**Affiliations:** UTHealth Houston, San Antonio, Texas, United States; UTHealth Houston, Houston, Texas, United States

## Abstract

Dementia caregiving presents unique challenges that may influence firearm storage behaviors, yet little research has examined how caregiver characteristics shape firearm storage practices. This study investigates firearm ownership, and firearm storage behaviors (i.e., storing firearms loaded/unloaded; locked/unlocked) among dementia caregivers using pooled data from the 2017, 2021, 2022, and 2023 Behavioral Risk Factor Surveillance System (BRFSS). The sample included 3,436 dementia caregivers (weighted N = 444,744), of whom 40.57% reported firearm ownership. Among firearm-owning caregivers (n = 1,460), 35.79% stored a firearm loaded, and 50.78% of those with a loaded firearm kept the firearm unlocked. Weighted logistic regressions revealed that male caregivers had twice the odds of keeping firearms loaded compared to female caregivers. Older caregivers (ref: < 50 year-old) were significantly more likely to store firearms unlocked. In contrast, caregiving for more than five years and assisting with household tasks were associated with safer storage (i.e., storing a firearm loaded; locked). Interaction analyses showed that firearm storage behaviors varied by gender, income, and caregiving intensity, with male caregivers in higher-income households and those with shorter caregiving durations being more likely to store firearms loaded and unlocked. Findings underscore the need for targeted firearm safety interventions for dementia caregivers, particularly among older and male caregivers, to mitigate risks associated with firearm access in dementia-affected households.

